# A model for oxygen conservation associated with titration during pediatric oxygen therapy

**DOI:** 10.1371/journal.pone.0171530

**Published:** 2017-02-24

**Authors:** Grace Wu, Alec Wollen, Stephen Himley, Glenn Austin, Jaclyn Delarosa, Rasa Izadnegahdar, Amy Sarah Ginsburg, Darin Zehrung

**Affiliations:** 1 Department of Biomedical Engineering, Boston University, Boston, Massachusetts, United States of America; 2 Consultant for PATH, Seattle, Washington, United States of America; 3 PATH, Seattle, Washington, United States of America; 4 Bill & Melinda Gates Foundation, Seattle, Washington, United States of America; 5 Save the Children, Fairfield, Connecticut, United States of America; University College London, UNITED KINGDOM

## Abstract

**Background:**

Continuous oxygen treatment is essential for managing children with hypoxemia, but access to oxygen in low-resource countries remains problematic. Given the high burden of pneumonia in these countries and the fact that flow can be gradually reduced as therapy progresses, oxygen conservation through routine titration warrants exploration.

**Aim:**

To determine the amount of oxygen saved via titration during oxygen therapy for children with hypoxemic pneumonia.

**Methods:**

Based on published clinical data, we developed a model of oxygen flow rates needed to manage hypoxemia, assuming recommended flow rate at start of therapy, and comparing total oxygen used with routine titration every 3 minutes or once every 24 hours versus no titration.

**Results:**

Titration every 3 minutes or every 24 hours provided oxygen savings estimated at 11.7% ± 5.1% and 8.1% ± 5.1% (average ± standard error of the mean, n = 3), respectively. For every 100 patients, 44 or 30 kiloliters would be saved—equivalent to 733 or 500 hours at 1 liter per minute.

**Conclusions:**

Ongoing titration can conserve oxygen, even performed once-daily. While clinical validation is necessary, these findings could provide incentive for the routine use of pulse oximeters for patient management, as well as further development of automated systems.

## Introduction

A reliable supply of oxygen is essential for treating hypoxemia, or low levels of oxygen in the blood, a major complication in many illnesses and conditions in infants, children, and adults [1–3]. In particular, oxygen is a life-saving treatment for hypoxemic children with pneumonia, the leading infectious cause of death for those under 5 years of age around the world [4]. The estimated 13.3% of children with pneumonia who have hypoxemia corresponds to 1.86 million cases each year [5]. Unfortunately, ensuring access to oxygen therapy is difficult in low- and middle-income countries, which have the highest disease burden [6, 7]. Such treatment may be required for several days [8], and a majority of health facilities in these settings (55%) have limited or no access to affordable, reliable oxygen supplies [9], so oxygen is often unavailable to patients who need it [10].

One way to address the lack of oxygen for therapy in low-resource settings could be oxygen conservation through routine titration. This is the process of adjusting delivered oxygen levels to maintain patient peripheral blood oxygen saturation level (SpO_2_) above the recommended target level of 90% at ≤ 2,500 meters (m) above sea level or 87% at > 2,500 m above sea level [5]. Titration is performed at the start of oxygen therapy to ensure that the patient is provided a sufficient flow of oxygen. Patients should also be monitored and titrated periodically since changes in SpO_2_ levels can occur. World Health Organization (WHO) clinical guidelines suggest that late desaturations, or hypoxemic episodes, can occur within an hour of titration [5]. Hyperoxemia, or excessively high SpO_2_, must also be managed to avoid overexposure and the resulting oxygen toxicity. Infants are especially susceptible to adverse effects from hyperoxemia, such as retinopathy [11]. To manage both hypoxemia and hyperoxemia, clinical staff should routinely monitor patient SpO_2_ levels. Previous estimates of oxygen consumed by pediatric pneumonia patients have not accounted for titration—except at the start of therapy—but rather, assumed that a fixed oxygen flow rate was needed at all times during treatment [8].

Pulse oximetry is a reliable and non-invasive technology that measures SpO_2_ and has become part of the oxygen therapy standard of care in well-resource settings, where it has been shown to be more effective than relying solely on clinical signs [10, 12]. This method requires less training than that needed when clinical signs are used to detect hypoxemia in children, an advantage where staff are often in short supply, over-burdened, and difficult to retain. In addition, the same pulse oximetry setup used for titration during oxygen therapy is used to diagnose hypoxemia. WHO clinical guidelines recommend that all children who are receiving oxygen administration be tested by pulse oximetry, when available, at least once a day and that it should be used regularly to monitor all children who show worsening respiratory distress, apnea, decreased consciousness, or any other clinical signs of deterioration [5].

Automated oxygen titration systems that include pulse oximeters have been developed recently; these systems help maintain patient SpO_2_ levels continuously within a target range without human intervention [13–15]. While not yet commercially available, they may offer a way to alleviate staff work burden. To better understand opportunities for use of these new systems—including automatic titration algorithms, product development, and resource allocation—we need a quantitative estimate of oxygen consumption over the course of treatment for pediatric patients with hypoxemic pneumonia.

In this study, we created a model of the oxygen needs of pediatric patients with hypoxemia, based on oxygen consumption data from previously published clinical studies [16]. The model, which assumed that all patients were started at an appropriate flow rate, was used to calculate the average amount of oxygen used in several scenarios: 1) no routine titration was performed; 2) routine titration was performed every 24 hours—a scenario for once-daily titration by a health care worker; and 3) routine titration was performed every 3 minutes—a scenario for continuous titration by an automated system.

## Methods

All modeling and simulation were performed using a custom script developed in MATLAB (MathWorks, Natick, MA, USA). Where possible, clinical data and guidelines specific to children with hypoxemic pneumonia were used to inform the model assumptions.

### Patient model

**Fig 1:**
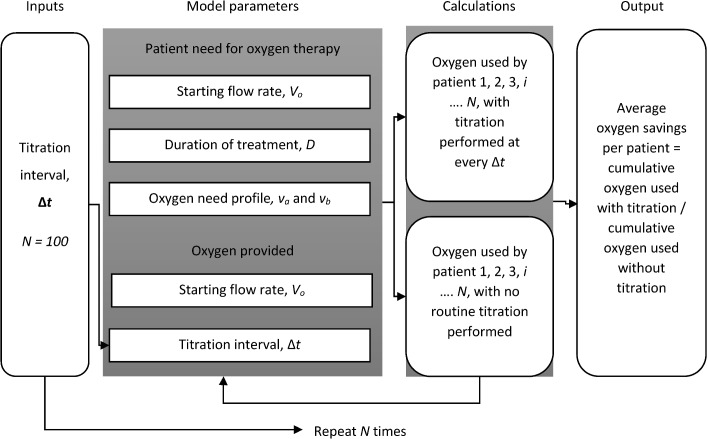
Overview of model and simulation used to estimate the oxygen usage and savings associated with routine titration during oxygen therapy of hypoxic children with pneumonia.

Fig 1 shows an overview of the model and simulation, which assume a source providing concentrated oxygen such as from an oxygen concentrator or a cylinder. The total oxygen used by an individual patient throughout the course of treatment, *O*_*2*,*i*_, was defined as a function of oxygen flow rate need over time, *V*_*i*_*(t)*, and the interval of time between titrations, Δ*t*.

$$O_{2,i}(\Delta t)=\sum_{t=0}^{D-\Delta t}V_{i}(t\times \Delta t)\times \Delta t$$

For this study, *t* was defined as time in days, and *D* was defined as the duration of treatment for patient *i* in days (see more below). The inputs used for Δ*t* are described below.

**Fig 2:**
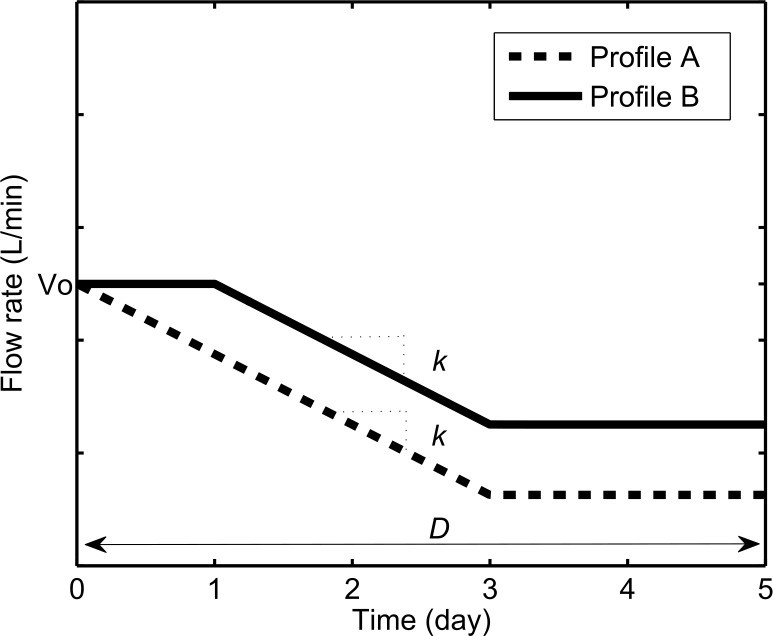
Two oxygen need profiles (Profile A and B, or v_a_(t) and v_b_(t), respectively) assumed to reflect patient oxygen requirements in order to maintain SpO2 > 90%.

For simplicity, patient needs for oxygen therapy, *V*_*i*_*(t)*, were randomly assigned to one of two profiles, *v*_*a*_*(t)* or *v*_*b*_*(t)* (Profile A or B as shown in Fig 2). Each profile was defined as a piecewise function, approximating the trends observed in previous studies reporting the flow rates provided to achieve SpO_2_ > 90% or 95% in children and infants with hypoxemia [16, 17]. Some patients were weaned off oxygen, or required less oxygen, as they improved over time. To reflect this weaning, a constant *k* was defined as 0.125 L/min/day based the study by Weber *et al* [16].

$$V_{i}(t)\in V,\;where$$

$$V=\{v_{a}(t),v_{b}(t)\}$$

P(V)=unif(V)

$$v_{a}(t)=f(x)=\left\{ \begin{array}{c} V_{0}+kt,\;0\leq t<3\\ V_{0},\;3\leq t<5 \end{array}\right.$$

$$v_{b}(t)=f(x)=\left\{ \begin{array}{c} V_{0},\;0\leq t<1\\ V_{0}+k(t-1),\;1\leq t<3\\ \;V_{0}+k(2),\;3\leq t<5 \end{array}\right.$$

To model the variability of patient oxygen requirements, a range of values and probability distributions were defined for the starting flow rate, *V*_*0*_, and length of treatment, *D*. WHO guidelines recommend that hypoxemic children should be started at between 1 and 2 L/min of oxygen and infants should be started at 0.5 L/min [1]. We assumed that starting flow rates occurred with equal probability. A study by Weber *et al* [16] reported the duration of oxygen therapy provided to hypoxemic children. Based on this, we defined the range of *D* to be between 1 and 5 days. We assumed that the corresponding probability distribution was a cumulative function of the reported data, normalized to the total number of patients receiving oxygen on the first day. Representative illustrations of pediatric pneumonia model patient needs for oxygen therapy are shown in Fig 2. Thus:

$$V_{0}=\{0.5,\;1.0,\;2.0\}\;where\;P(V_{0})=unif\{V_{0}\}$$

$$D=\{1,2,3,4,5\}\;where\;P(D)=\left\{ \begin{array}{c} 0.32,\;for\;D=1\\ 0.27,\;for\;D=2\\ 0.18,\;for\;D=3\\ 0.12,\;for\;D=4\\ 0.11,\;for\;D=5 \end{array}\right.$$

The key assumption of this model was that patients were first started on an appropriate flow rate that maintained SpO_2_ levels within the target range. In addition, we assumed that SpO_2_ levels immediately were brought within the target SpO_2_ range upon titration, and that no subsequent desaturations occur.

### Simulation outputs

To estimate more accurately the average oxygen savings per patient, the model was iterated *N* times, where *N* represents the number of patients admitted to a health facility and requiring oxygen therapy. In doing this, the cumulative oxygen usage of *N* patients was calculated for each Δ*t*. For our study, *N* was evaluated for 100 patients. Thus:

$$O_{2,\;total}(\Delta t)=\sum_{i=1}^{N=100}O_{2,i}$$

The output of this simulation was a calculation of the average oxygen savings, defined as the ratio between cumulative oxygen consumed when routine titration was performed and cumulative oxygen consumed when routine titration was not performed. Thus, for a given Δ*t*_*i*_:

$$Oxygen\;savings=\frac{O_{2,total}(\Delta t)}{O_{2,total}(D)}$$

Results are reported as the average ± the standard error of the mean for three repetitions of the simulation.

### Use case scenario assumptions

Oxygen usage and savings were calculated for three scenarios by adjusting the input Δ*t*. In Scenario I, the patient was prescribed *V*_*0*_ and no routine titration was performed during the course of oxygen treatment. Here, Δ*t* = *D*, and the patient was assumed to be given *V*_*0*_ for the full length of *D*. Results from Scenario I served as the study reference. In Scenario II, the patient was monitored every 24 hours by a health care worker and the oxygen flow rate adjusted manually (Δ*t* = 24 hrs). In Scenario III, the patient was assumed to be monitored and oxygen flow rate adjusted every 3 mins by an automated pulse oximetry titration system (Δ*t* = 3 mins).

## Results

### Oxygen usage and volume saved with titration

**Fig 3:**
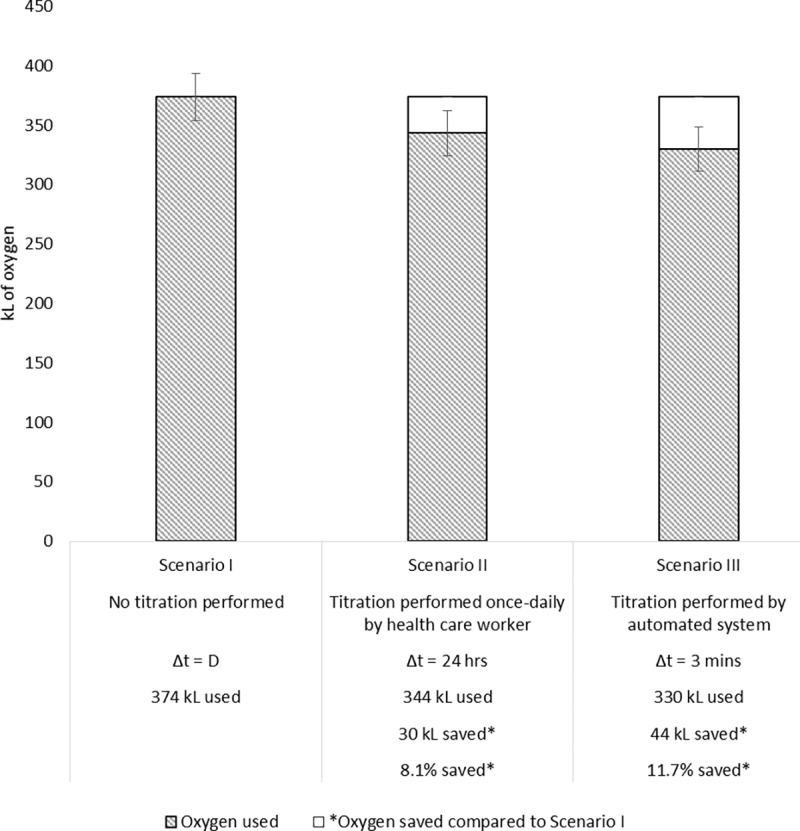
Cumulative oxygen usage for 100 patients, in scenarios with three different titration intervals. Δt is the titration interval.

Using the model, we assessed the oxygen usage of 100 patients for three titration scenarios: no titration, titration every 24 hours, and titration every 3 minutes (Scenarios I, II, and III, respectively). As shown in Fig 3, when no routine titration was performed, 374 ± 20 kL of oxygen were used. When titration was simulated every 24 hours on the same hypothetical set of patients, 344 ± 19 kL of oxygen were used. When titration was performed every 3 minutes, 330 ± 19 kL of oxygen were used. As a result, 30 kL and 44 kL of oxygen were saved in Scenarios II and III, respectively, compared with Scenario I. The 30 kL of oxygen saved with routine titration every 24 hours is equivalent to the volume delivered via a standard flow of 1 L/min of oxygen for 500 hours. Similarly, 44 kL of oxygen saved by titrating every 3 minutes is equivalent to 733 hours of oxygen therapy.

This average oxygen saved per patient was 300 L and 440 L, or 8.1% ± 5.1% to 11.7% ± 5.1%, for titration every 24 hours and 3 minutes, respectively. These savings also represent the minimum and maximum possible within this range of titration intervals.

## Discussion

Based on our modeling analyses, a simple routine of once-daily titration conserves oxygen during oxygen therapy for pediatric pneumonia patients. When compared with a scenario where no routine titration is performed and the patient is provided oxygen at a fixed flow rate for the duration of treatment, once-daily titration provides an estimated 8.1% ± 5.1% in oxygen savings (Fig 3). Performing titrations more than once per day would conserve more oxygen, although our results suggest that savings would not exceed 11.7% ± 5.1%. In other words, once-daily titration achieved more than two-thirds of the oxygen savings achieved via continuous titration—meaning every 3 minutes in our model. The cumulative savings from conserved supplies are considerable when multiple patients need oxygen simultaneously: this conservation would provide 5.0 to 7.3 hours of additional oxygen treatment per patient, prescribed at 1 L/min.

The estimated 8% to 12% range of oxygen savings found in this study was not as high as that provided by some other oxygen conservation techniques. Delivery-on-demand mechanisms, which provide boluses of oxygen rather than a continuous flow, theoretically can provide a savings ratio of up to 7:1, a savings of approximately 86% [18]. Transtracheal catheters were noted to provide up to 3:1 savings (approximately 66% savings). Similarly, reservoir cannulas reportedly provide up to 4:1 savings (75%) of oxygen, although these are currently commercially available only for adult use [18]. In spite of these impressive theoretical savings, observed savings in clinical use of these devices in children are often reported. One clinical study of synchronized demand-on-delivery mechanisms demonstrated a theoretical and an observed savings ratio of 1.9:1 (nearly 50%) [19].

### Automated titration systems

Near-continuous titrations (e.g., every 3 minutes) theoretically conserve the maximum amount of oxygen possible. However, manual titration by nursing staff at this rate is not feasible. An automated closed-loop titration system that maintains patient SpO_2_ levels within a target range could be beneficial, especially in reducing the risk of oxygen toxicity in preterm infants. In addition, desaturations can be addressed quickly, potentially improving clinical outcomes and freeing up nursing time for other health care activities, an important consideration in low-resource settings where staff resources are limited [20–22]. Such systems require a titration control algorithm integrated with mechanisms for both oxygen delivery and monitoring [13]. Algorithms to control the timing, magnitude, and frequency of titration are challenging to develop and require careful validation to maximize the time during which patients are within the target SpO_2_ range. Recent efforts in developing algorithms for non-mechanically ventilated neonates show promise in clinical studies [23], but more studies are needed for infants and children.

### Advantages of pulse oximetry

Pulse oximetry is the current gold standard for monitoring when to start, stop, and adjust oxygen therapy [1]. These medical devices are relatively affordable and easy to use and require less training than detection of clinical signs. In some health facilities of low- and middle-income countries, insufficient equipment, capital, staff, access to replacement parts, and awareness result in low usage [24, 25]. As a substitute, oxygen therapy is sometimes administered based on clinical signs of hypoxemia, such as grunting respiration, cyanosis, and head nodding [26, 27]. However, these signs are nonspecific, and can present in other common illnesses that affect children. One study suggested that diagnosis based on clinical signs would have resulted in missed hypoxemia diagnoses in some cases, and inappropriate oxygen prescription in others, for approximately 30% of admitted children admitted to rural hospitals in Papua New Guinea [10].

Promoting awareness and providing incentives could increase the use of pulse oximeters to guide oxygen therapy in low-resource settings. In addition to its role in saving oxygen via titration, pulse oximetry—along with integrated management of pneumonia—could be highly cost-effective, costing as little as $2.97 per disability adjusted life year (DALY) averted, as modeled by Floyd *et al* [28]. This cost also accounted for battery replacements, which are sometimes problematic in low-resource settings [29, 30].

### Study limitations

The limitations of our model stem primarily from the lack of clinical data, which are required to predict SpO_2_ levels based on the fraction of inspired oxygen (FiO_2_) levels. FiO_2_ levels can be adjusted by the flow rate of delivered oxygen and mode of delivery (e.g., face masks, cannulas, or catheters), but can also be influenced by a number of factors such as the patient ventilation rate and oxygen concentration (both ambient and of the delivered flow) [31–33]. The most detailed study available analyzed the flow rates needed to achieve SpO_2_ > 95% in 110 hypoxemic children and infants over 7 days in The Gambia [16]. Results from similar studies demonstrated comparable trends, although data were reported for only 3 instead of 7 days of observation [17]. While it is generally understood that SpO_2_ levels increase in response to greater FiO_2_ delivery, more precise predictions would be needed to refine future model predictions. Other than FiO_2_ delivery, SpO_2_ levels can also be influenced by human factors that disrupt the flow of oxygen to the patient. For example, patient movement in infants can result in obstruction of the airways, resulting in hypoxemia. Such factors likely vary between patients and were not included in this model.

The overall duration of oxygen therapy in a no-titration scenario may be shorter or longer than in the presence of routine titration or monitoring. When oximetry is available, it should be used not only for initial and routine titration, but also for determining when to initiate and discontinue therapy. Therefore the savings in this model do not account for the potential overall effect of routine monitoring on a single patient’s treatment course. The model also does not account for the potential overall effect on the treatment ward. For example, more or fewer children may be started when oximetry is used as the indication to start rather than by clinical signs.

Another limitation of the model was that we assumed that patients always received sufficient oxygen to immediately maintain SpO_2_ levels within a target range. This may not be realistic, as the SpO_2_ response times to FiO_2_ changes can take a few seconds to minutes [34, 35] and can vary depending on whether pulse oximetry or clinical signs are used. Furthermore, both rapid and slow changes in oxygen saturation can occur even if SpO_2_ levels appear stable at first. Desaturations can occur during oxygen therapy—for example, during anesthesia [36] and in intensive care [15, 34]. The frequency and duration of desaturations have not been well studied but appear to vary with age [37] and oxygen delivery method, at least [38]. If desaturations occur during pediatric hypoxic pneumonia treatment, our analyses may have overestimated oxygen savings, because more oxygen may be needed to bring the patient’s saturation level back into normal range.

In spite of these limitations, our simple model based on available clinical data provides the first estimates of oxygen savings associated with titration. Our model could be combined with findings from other models, such as that developed by Bradley *et al*.[8], which predicts total oxygen needs in a health facility given the seasonality of pneumonia, prevalence of hypoxemia in pneumonia patients, and oxygen flow rates (0.5 to 1 L/min) required to maintain oxygen saturation above recommended levels.

### Future directions

The results from this study on oxygen savings associated with titration using pulse oximetry can be used by product developers in designing appropriate technologies, especially for low-resource settings. To refine the model, more clinical data are needed to predict SpO_2_ levels in response to oxygen therapy. This includes the influence of flow rate (or delivered FiO_2_), target SpO_2_ ranges, methods of titration (pulse oximetry or clinical signs), and altitude. Systematic issues should also be addressed, such as increasing awareness of safe oxygen use and availability of pulse oximetry. Automated controllers of inspired oxygen concentration could be a promising technology to reduce adverse events from hypoxemia and hyperoxemia.

## Conclusions

Based on modeling estimates, once-daily titration during oxygen therapy could conserve oxygen, with more frequent titration yielding greater savings. This conservation would complement the safe use of oxygen that is provided with clinical supervision and proper modes of routine monitoring tools, such as pulse oximetry. In particular, titration could benefit health facilities in low-resource settings where oxygen supplies are often limited. Further clinical validation of pulse oximetry for patient management can build upon the findings from this evaluation.

## Supporting information

S1 File Titration Model MATLAB Script. (M)

S2 File Flow Calculation Helper Function. (M)
